# Editorial: Hormonal Regulation of Non-climacteric Fruit Development and Maturation

**DOI:** 10.3389/fpls.2021.690691

**Published:** 2021-05-25

**Authors:** Vanessa Galli, María Teresa Sanchez-Ballesta, Ashraf El-kereamy, Ricardo Antonio Ayub, Wensuo Jia

**Affiliations:** ^1^Technology Development Center, Federal University of Pelotas, Pelotas, Brazil; ^2^Department of Characterization, Quality and Safety, Institute of Food Science, Technology and Nutrition (ICTAN-CSIC), Madrid, Spain; ^3^Department of Botany and Plant Sciences, University of California, Riverside, Riverside, CA, United States; ^4^Laboratory of Biotechnology Applied to Fruticulture, Ponta Grossa State University, Ponta Grossa, Paraná, Brazil; ^5^College of Agronomy and Biotechnology, China Agricultura University, Beijing, China

**Keywords:** non-climacteric, phytohormones, fruit disorders, ripening (fruit), abiotic stress, crosstalk

Non-climacteric fruits, such as strawberry, grape, raspberry, cherry, citrus, and many others, are characterized by ripening transitions that do not strictly depend on a significant increase in ethylene production and an associated rise in respiration rate. The development and maturation processes of these fruits follows a series of molecular and physiological events that leads to modifications in size, color, texture, flavor, and aroma. Although the ripening process of climacteric fruits has been well-documented, the signals triggering in non-climacteric fruit remains poorly understood. However, the involvement of phytohormones in this process has been demonstrated. The current Research Topic on “Hormonal Regulation of Non-climacteric Fruit Development and Maturation” is a combination of research articles and review articles, providing novel insights and detailed overviews on the current knowledge of different aspects of non-climacteric fruit ripening and the metabolites affected by phytohormones.

Abscisic Acid (ABA) is considered to play important roles in non-climacteric fruit ripening. This phytohormone has been associated with the improvement of anthocyanins content, especially in strawberries and grapes. However, the effect of ABA in other ripening aspects is still poorly explored in the literature. By taking advantage of the availability of a fruit-specific ABA-deficient mutant named Pinalate from the Navelate orange [*Citrus sinensis* (L.) Osbeck], Romero and Lafuente investigated the implication of ABA in the citrus ripening program, particularly affecting cuticle properties and epicuticular wax metabolism, composition, and morphology. Through a series of morphophysiological, metabolites and molecular analysis, the authors demonstrated that ABA has an influence on cuticle permeability by altering wax metabolism and morphology. This study contributes to a better understanding of ABA's effect on wax and, hence, for future approaches aiming at reducing the susceptibility to detached fruit dehydration.

Although ethylene has been considered the play master in the ripening of climacteric fruits, its contribution to non-climacteric ripening has been investigated. Through the exogenous application of ethephon and the ethylene-action inhibitor 1-methylcyclopropene (1-MCP) in strawberry attached to the plant, Reis et al. show evidence that ethylene does not play a key role in strawberry ripening as it did not change the rate of ripening. However, the authors pointed out specific aspects of fruit development and metabolism that were affected by ethephon, which was dependent on the stage of fruit development, and argued that these effects were imparted by a stress response.

Besides the contribution of phytohormones in the ripening of non-climacteric fruits, the signaling cascade following the induction of phytohormones and the whole metabolic changes necessary toward the induction of this phytohormone-dependent ripening is still poorly characterized. In this regard, Chervin discussed the contribution of starch accumulation or breakdown for the definition of climacteric and non-climacteric fruits. This intriguing review makes us consider and reconsider the fundamental aspects differentiating these two fruit classes and how much we still do not know about fruit ripening and carbohydrate metabolism. Besides the carbohydrates, the role of polyamines in the phytohormone-dependent induction of ripening at the physiological and molecular levels has been considered and was explored by Gao et al. This review provides a deeper understanding of the metabolism and regulatory roles played by polyamines in climacteric and non-climacteric fruit ripening and pointed out the interplay among these metabolites and phytohormones; signaling molecules, such as H_2_O_2_, nitric oxide, and Ca^2+^; and nitrogen signaling. The repercussions of studies in this area may help put another piece in the puzzle, which is the understanding of ripening.

Finally, this Research Topic brings the studies of Ribalta-Pizarro et al. and Jia et al. Through omics researches it has been possible to observe that the metabolism and signaling pathways during the ripening process are similar to those affected during osmotic stresses. Some authors have argued that osmotic stresses trigger the signaling pathway toward the ripening process. In this context, through several water status, water, and potential osmotic parameters, Jia et al. have demonstrated how the osmotic potential can contribute to fruit ripening initiation in strawberries. The change in fruit water potential is most likely caused by the catabolism of large molecules in receptacle cells and the involvement of osmotin-like proteins (OLPs); this decrease in fruit osmotic potential then triggers the onset of fruit ripening. Moreover, Ribalta-Pizarro et al. showed that small changes in water supply through irrigation could affect grape growth and quality. By studying the whole fruit and tissue-specific content of several phytohormones, notably ABA, ethylene precursor, jasmonic acid, salicylic acid, auxins, and cytokinins, the authors provide a view of how these hormones vary along fruit ripening and how and if they are affected by the reduced water supply. Taken together, the studies of Jia et al. and Ribalta-Pizarro et al. provide evidence for the influence of water status on the induction of fruit ripening mediated by hormones.

Given the increased world population, understanding the major players in the ripening process is necessary to develop novel approaches aiming to improve agricultural practices, food security, and minimize losses during harvest, transport, and storage. We hope that the reader will find a useful reference for the state of the art in the hormonal regulation of the non-climacteric ripening process in this Research Topic.

## Author Contributions

VG wrote the first draft. All authors listed have made substantial contribution and approved the manuscript for publication.

## Conflict of Interest

The authors declare that the research was conducted in the absence of any commercial or financial relationships that could be construed as a potential conflict of interest.

